# Food for thought: Perspectives on the current state of Vertical Farming

**DOI:** 10.1038/s44319-025-00518-1

**Published:** 2025-07-21

**Authors:** Jeremy Harbinson, Craig R Taylor

**Affiliations:** 1 Laboratory of Biophysics, Wageningen University, Stippeneng 4, 6708 WE Wageningen, The Netherlands; 2 Burgemeester Verheugtstraat, 5731 AJ Mierlo, The Netherlands

**Keywords:** Economics, Law & Politics, Evolution & Ecology, Plant Biology

## Abstract

The slew of bankruptcies in sustainability-focused vertical farming brings its own future sustainability into focus. Addressing scientific and economic obstacles will be key to its future prospects.

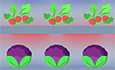

Barely 10 years ago, it was not uncommon to read about another vertical farm commencing operations in an abandoned building in cities in the USA or Europe. A new agricultural paradigm was taking root: ‘Dirty’ and declining city centres were no longer a problem, but rather part of the solution for food production, and seemingly anyone with access to a building—often abandoned ones—could participate in the revolution. At face value, the idea of vertical farms in urban centres was attractive for all stakeholders: growers had the opportunity to transform the conventional agricultural system—often criticised for its high-carbon footprint and liberal use of pesticides—into what was claimed to be a cleaner, more efficient and sustainable food production paradigm. Often located within densely populated areas, the proximity of vertical farms to end consumers would drastically reduce lengthy and fuel-consuming transport across state or country borders. City administrators could incorporate vertical farms into urban renewal programs, replacing derelict buildings in once vibrant city centres with this new green, environmentally friendly agricultural model. Venture capital with an appetite for ‘green’ projects sensed the opportunity to make healthy returns in what promised to be an exciting shift in the *status quo* of fresh-produce cultivation.

‘Dirty’ and declining city centres were no longer a problem, but rather part of the solution for food production, and seemingly anyone with access to a building—often abandoned ones—could participate in the revolution.

It is often the case that new technologies experience a surge of early-market adopters followed by a subsequent reduction in the number of participants as a result of competition for market share, inadequate funding, inability to acquire the necessary knowledge quickly enough or simply by the vagaries of free markets. With the recent slew of bankruptcies, including former frontrunners in the sector with once healthy funding, it seems that vertical farming (VF) has reached and surpassed this point, but it also reveals unforeseen challenges and the need for recalibrating early expectations surrounding what remains a sector in its infancy. It is important nonetheless to preface this article by acknowledging the headwinds which any emerging sector can be expected to face, but also to note that this is a useful juncture in the progression of the vertical-farm sector to explore some of the key challenges and considerations facing VF and which opportunities can be harnessed to realise its potential.

## Vertical farms

**Figure 1:**
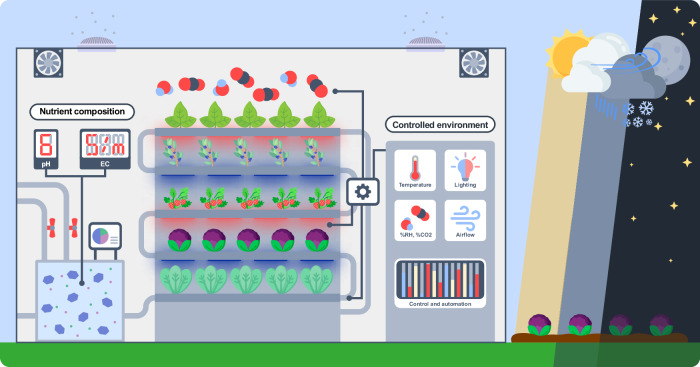
Protected from the elements. A typical implementation of a vertical farm, characterised by stacked layers of plants and lighting in a highly controlled environment.

We first take a look at what a vertical farm actually is. There can be many definitions and indeed many permutations, but at its heart, it is, as the name suggests, any plant production system in which crops are grown in multiple layers at different heights. In contrast, one can think of conventional agriculture in the field or greenhouse as a 2D production format, whereas VF uses a three-dimensional format. In fact, even certain greenhouse production systems where leafy crops are produced in vertical towers or where strawberries are produced in gutters at different heights could meet this broad definition of VF. However, a far more common implementation is the vertical stacking of independent plant production layers, much like how a multi-storey building comprises multiple independent floor spaces (Fig. 1). A vertical farm is also enclosed within a building and driven by the same goal of maximising usable space using the lowest possible footprint.

Quite obviously, a building shell on its own cannot support plant life—it can only provide a space which is buffered from the outside and where all input and output fluxes relevant to plant growth must be supplied and carefully controlled in a predominantly closed production system. These fluxes include water, nutrients and CO_2_-enriched air, and the composition and properties of each—for instance water chemistry, gas composition, air speed or water vapour—needs to be monitored and controlled. Light deserves special mention since the space efficiency gained by stacking vertical layers inevitably comes at the expense of natural light. While some designs have managed to incorporate natural daylight by using a transparent roof and cascading plant layer arrangements, most VF designs require artificial light as sole-source lighting. The combination of lighting, monitoring and control technologies is central to the definition and operation of a VF.

Even a superficial description of a vertical farm makes evident the high degree of environmental control and predictability compared to outside ambient conditions. Traditional forms of agriculture—open fields and greenhouses—are both subject to the vagaries of weather to a greater and lesser extent, respectively. Generally, changeable environmental conditions are not desirable for plant growth as they can create stress by drifting outside desired tolerances, inducing acclimation mechanisms which may not fully compensate for reductions in growth. For as long as modern agriculture has existed, growers have sought to ameliorate the effects of undesirable weather or climate on their crops. Open-field growers routinely use wind breaks to buffer crops from wind, shade netting to temper the harsh sun or protect against hail, water sprays to protect against frost, and irrigation systems to avoid water stress. The modern greenhouse dates back about 200 years, and they extended the growing season into parts of the year which were otherwise too cold for the cultivation of warm-season crops. Modern production greenhouses are unrecognisably more sophisticated than their early counterparts, with computer-controlled heating and cooling, irrigation, CO_2_ enrichment and, in some cases, LED lighting. Still, greenhouses are not buffered entirely from ambient conditions—for example, incoming solar radiation and spectrum are highly variable.

**Figure 2:**
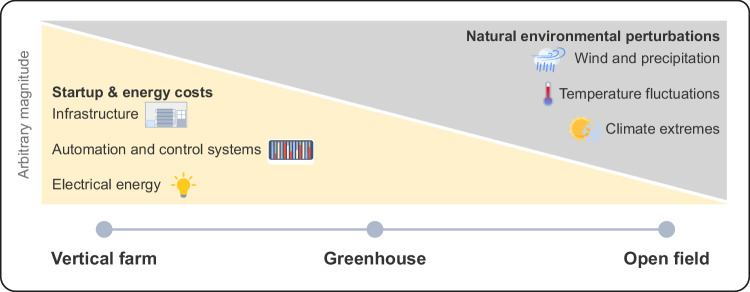
The cost of control. The inverse relationship between the magnitude of start-up and energy costs of plant production systems, such as a vertical farm, a greenhouse or an open field, and the degree to which it is protected from natural environmental perturbations.

If all that mattered were precise, predictable and repeatable growing conditions, then VF is undoubtedly the state-of-the-art model of agriculture in the progression of increased independence from weather conditions. The ability to maintain a tightly controlled climate means that environmental variables can be adjusted to maximise growth and yield. However, it is an inescapable reality that VF forms part of a highly competitive and generally low-margin food chain which, in many parts of the developed world, is robustly subsidised. The key challenge facing VF is how to be economically viable against this backdrop while also facing high costs associated with automation and a high level of control of growth conditions. We expand more fully on some of the major input costs but prime the reader with a generalised illustration of the relationship between input costs and level of environmental control of VF compared to greenhouse and open-field production (Fig. 2).

The ability to maintain a tightly controlled climate means that environmental variables can be adjusted to maximise growth and yield.

The high start-up and energy costs of vertical farms are evidenced by two observations: first, the widespread tendency of VF to limit production to only high-harvest index, high-value leafy crops such as culinary herbs and, second, the alarming number of once well-financed vertical farms around the world which have in the recent past gone into liquidation. The general picture which emerges from these observations is that vertical farms are currently struggling to compete with traditional agriculture on profitability. Exploring where gains in system efficiency can be made is of paramount importance to make the VF model competitive and financially feasible.

## Light supply

Multiple energy transductions occur within a vertical farm, and it is the result of these efficiencies which determines total system efficiency. The most significant energy transductions are the conversion of electrical energy to light energy, which is subsequently converted to chemical energy by photosynthesis. A fundamental difference between VF and conventional agriculture is that energy input represents a significantly higher fraction of the overall input costs, and lighting is a major part of these energy costs. While some greenhouses in higher latitudes employ supplementary lighting in the autumn-winter-spring period, this is, as the name suggests, not the sole source of lighting as is the case with VF, and it is often not needed during the summer months when the intensity and duration of natural daylight are sufficiently high.

A fundamental difference between VF and conventional agriculture is that energy input represents a significantly higher fraction of the overall input costs, and lighting is a major part of these energy costs.

**Figure 3:**
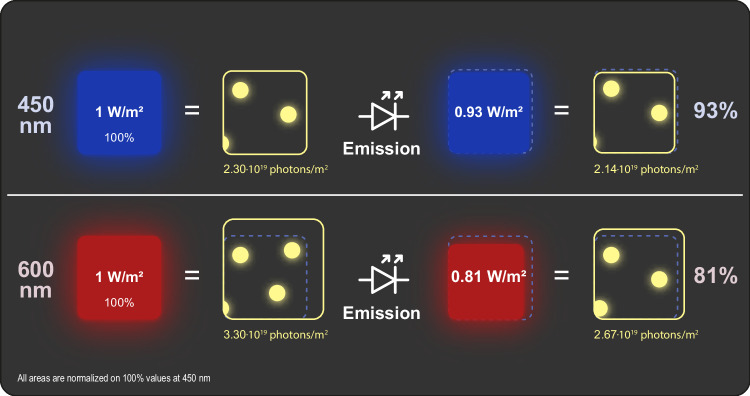
A comparison of the electrical efficiencies of photon emission by red and blue LEDs. Despite blue LEDs being more electrically efficient than red LEDs in energy terms (93% compared to 81% in terms of Watts of light emitted per square metre per Watt of electrical input), their emitted light energy is divided between fewer, higher-energy, photons compared to red light. The result is that more photons are emitted by the less efficient red LEDs because the energy content of red LEDs is less than that of blue LEDs. With the number of photons being more relevant to photosynthesis rather than absolute energy per photon, red LEDs are preferred over blue LEDs from a quantum (or photon) production perspective.

LEDs are the standard for lighting, given that their efficiency is greater than other technologies. In addition, they are relatively cheap, compact and long-lived, and modern LEDs are now at a point where their theoretical maximum efficiencies are within reach: red (660 nm) and blue (450 nm) LEDs have efficiencies of 81% and 93%, respectively, in terms of Watts of light emitted per unit Watt of electricity supplied (Fig. 3; Kusuma et al, 2020). From a lighting efficiency perspective, therefore, there is not much room for improvement, at least if losses arising from the design of the LED packages and light fixtures themselves are not taken into account (Kusuma et al, 2020). Therefore, any more sizeable increases in VF efficiency will need to be sought in other areas, such as how efficiently photosynthesis utilises light to drive carbon dioxide assimilation and subsequent production of energy-rich carbohydrates.

Photosynthesis can be divided into two stages. The first is the absorption of light by photosynthetic pigments and the conversion into a metabolically useable form—ATP and NADPH—in the so-called ‘light reactions’. The second is the use of this chemical energy to convert carbon dioxide into carbohydrates in the leaf chloroplasts: the ‘dark reactions’. While leaf photosynthesis uses light in the wavelength range of 400 nm to about 730 nm (violet to red), the range of photosynthetically active wavelengths is usually defined as 400 to 700 nm. This difference is due to the fact that wavelengths above 700 nm are only poorly used for photosynthesis when applied alone, but these longer wavelengths are more effectively used when applied together with other wavelengths, which is normally the case (Taylor et al, 2025). Leaf absorption of light in the 400–730 nm wavelength range is up to 95% in the blue and red spectral regions, but lower, about 70%, in the green part of the spectrum, which is why plants are green.

**Figure 4:**
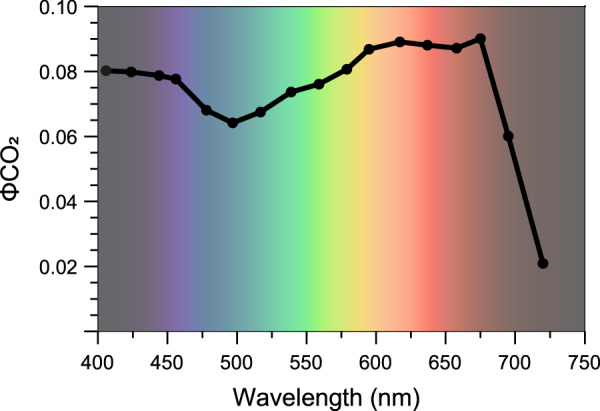
The light-use efficiency of CO_2_ fixation in tomato leaves on an absorbed light basis for each of 17 narrowband irradiances between 406 and 720 nm (data from Taylor et al, 2025). These light-use efficiency values were obtained using strictly light-limiting irradiances and non-photorespiratory conditions so these efficiencies are the maximum that can be obtained for each of these wavelengths.

The process of converting a single carbon dioxide molecule to carbohydrate requires 3 ATP and 2 NADPH—the latter equivalent to 4 reduced ferredoxins. The textbook light-use efficiency (LUE) for carbon dioxide fixation is 0.125 moles of carbon dioxide fixed per mole of photons in the 400–700 nm wavelength region. This is based on the simplifying assumptions that carbon dioxide assimilation is limited by the supply of NADPH and that photosynthesis is perfectly efficient in terms of the use of absorbed light. In practice, however, the maximum measured LUE on an absorbed light basis for carbon dioxide fixation is somewhat less than 0.125—typically around 0.090 in the red wavelength region (Fig. 4; Hogewoning et al, 2012; Taylor et al, 2025). This lower value reflects the inefficiencies of photosynthesis and other physiological complications, such as the use of ATP and ferredoxin or NADPH for metabolic processes apart from carbon dioxide fixation and the need for processes such as cyclic electron transport.

In addition, maximum LUE values of around 0.090 are measured under conditions that eliminate another major inefficiency of photosynthesis—the process known as photorespiration. The first step of the metabolic engine of photosynthesis is the reaction between ribulose-1,5-bisphosphate (rubp) and carbon dioxide, catalysed by the enzyme ribulose-1,5-bisphosphate carboxylase oxygenase, which mercifully, is generally just called rubisco. This enzyme catalyses, however, not only the carboxylation of rubp by carbon dioxide but also its oxygenation by O_2_. The latter reaction takes place at a significant rate in most leaves in air and competes with the carbon dioxide fixation process to such an extent that eliminating the oxygenation reaction under the conditions typically used to measure photosynthesis increases the LUE of carbon dioxide fixation by about 50% under typical measurement conditions. Suppressing oxygenation can be achieved by increasing the carbon dioxide concentration or decreasing the oxygen concentration. The former is more practical: in commercial greenhouses, the carbon dioxide concentration is typically 800–1000 ppm, which increases the rate of carbon dioxide fixation and therefore plant growth. The rate of the oxygenation process relative to that of carboxylation is also strongly temperature-dependent, increasing with increasing temperatures.

From a photosynthetic perspective, all absorbed photons with an energy above the quantum energy threshold of a red photon are able to drive photosynthesis equally well, or at least almost equally well. The photosynthetic LUE of blue and green photons is lower than that of red, yellow and orange photons because of the inefficiency of energy transfer from photosynthetic carotenoids to chlorophyll, and the presence of non-photosynthetic pigments such as flavonoids that compete with photosynthetic pigments for light. These non-photosynthetic pigments are not necessary in vertical farming and could be dispensed with—a low-hanging fruit for crop improvement.

Once absorbed by a chlorophyll—or transferred to a chlorophyll from a carotenoid pigment—the quantum efficiency with which these photons are used for photochemistry can be up to 99% in photosystem I and 90% in photosystem II under optimal conditions, that is, red photons under strictly light-limiting conditions, and with a balanced excitation of both photosystems. If we exclude the loss of efficiency due to carotenoids and non-photosynthetic pigments, this efficiency is the same for a more energy-rich blue photon as it is for a red photon—the extra energy in the blue and other short-wavelength photons is simply lost as heat once they are absorbed by the photosynthetic pigments. This has consequences for plant lighting. While blue LEDs are more energy efficient than their red counterparts, they still produce 35% fewer photons per unit electric energy, and each blue photon has energy in excess of what can be trapped by photosynthesis; note that this excess energy is not the same as the excess light energy referred to in connection with photodamage. With the number of absorbed photons—rather than the total energy carried by these photons—being more important for photosynthesis (it is a quantum process), red LEDs are more efficient for driving photosynthesis (Fig. 3).

This discussion on the merits of red light could easily lead to the assumption that red light alone is all that is needed in VF. However, longer-term exposure to only red light has been shown to induce a peculiar array of physiological disorders in some crops, aptly named the ‘red-light syndrome’ (Trouwborst et al, 2016). This includes unresponsive stomata and impaired photosynthesis. Thankfully, these anomalies are easily remedied by the addition of even small amounts of blue light. More generally, this phenomenon highlights the importance of different colours of light, which serve not only as an energy source but also as a source of valuable information about the plant’s immediate environment. Far-red rich irradiance, for example, tends to induce the ‘shade avoidance syndrome’ which most notably involves stem elongation in an adaptive attempt to outcompete neighbouring plants at reaching and capturing light energy. Some of the adaptive responses to light composition are probably more relevant in the wild than in the safe haven of a vertical farm where excessive competition is discouraged by suitable planting densities and crop management. Specific lighting recipes may nonetheless be exploited to create plants which are either more or less compact for the purposes of a specific production layer height, better shelf life, or simply to create a morphology, colouration or flavour with greater consumer acceptance.

**Figure 5:**
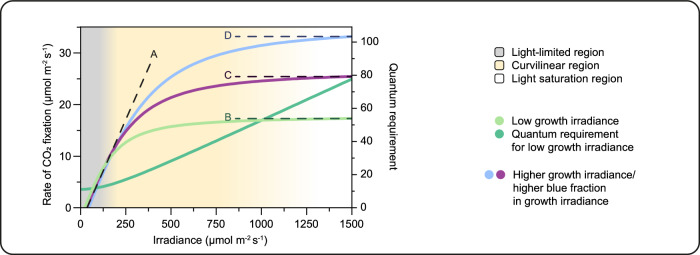
Example of three different light response curves (light green, purple, and blue) showing the relationship between CO_2_ fixation and light intensity, each with a different maximum photosynthetic capacity (dashed lines B, C, and D). The maximum photosynthetic capacity differs between species and genotypes and is influenced by growth environment; a higher growth light intensity or a higher blue-light fraction in growth irradiance can, within limits, increase the maximum capacity. Note, however, how the initial slope of each light response curve is relatively invariant from dashed line ‘A’, which represents the maximum light-limited slope of CO_2_ fixation, indicating similar light-limited light-use efficiencies despite large differences in maximum photosynthetic capacity. The lack of sharp transitions between the light-limited, curvilinear, and light-saturated regions of these curves is due to the continuous increase in the quantum requirement for CO_2_ fixation once light intensity is increased beyond the zone of strict light-limitation (shown here in dark green for curve B, the example with the lowest maximum rate of CO_2_fixation). The relationship between carbon fixation and irradiance is very flexible and responsive to the environment (irradiance, temperature, nutrition and water supply and stomatal regulation) as well as internal factors such as leaf age, leaf and plant history and plant genotype.

Another problem is that the maximum LUE for carbon dioxide fixation can be achieved only over a range of low-light intensities, but these low-light intensities produce only a low rate of photosynthesis (Fig. 5). Increasing the light intensity results in a progressive decrease in the LUE of carbon dioxide fixation; that is, an increasing number of photons is needed per carbon dioxide fixed. So, while increasing light intensity increases the amount of photosynthesis, it does so with increasing cost in terms of the number of quanta required per unit of carbon dioxide fixed. While the maximum LUE of carbon dioxide fixation is believed to be relatively invariant between plants because it is based on a common photosynthetic engine, the degree to which LUE decreases with increasing irradiance does vary strongly between genotypes and species. The decrease in photosynthetic LUE with increasing irradiance is paralleled by an increase in thermal dissipation of the absorbed light and the curvilinear photosynthesis/light-intensity relationship, which eventually becomes light-saturated. The efficiency with which leaves utilise higher light intensities is a highly plastic trait which shows increases in response to higher light intensity during growth and, usually to a lesser degree, after full leaf expansion. This response is accompanied by a higher maximum photosynthetic capacity, though the ability of leaves to increase their photosynthetic capacity in response to an increased irradiance is finite. The spectrum of the light is also important: an increased fraction of blue light in the growth spectrum will tend to increase LUE at higher light intensities by increasing the maximum rate of photosynthesis the leaf can achieve (Hogewoning et al, 2010), but blue photons are more expensive in energy terms than red. There is therefore a complex physiological and environmental management relationship to maximise profit from control of light, and this optimum operating point will also vary from genotype to genotype.

The properties of photosynthesis summarised above strongly influence the way that expensive light is supplied to plants in VF systems: it is about the efficient generation of a photosynthetically active quantum flux combined with operating at a high plant LUE, including whole-plant light absorption. Photons in the red and blue regions of the spectrum are both strongly absorbed by leaves but the yield of red photons at 660 nm operating with an energy efficiency of 0.81 W W^−1^ is 4.5 μmol s^−1^ W^−1^, whereas for blue photons at 450 nm operating with an energy efficiency of 0.93 W W^−1^ the photon yield is 3.5 μmol s^−1^ W^−1^. Lighting for VF systems therefore usually use predominantly red LEDs, and while a 680 nm red LED would be closer to the leaf light absorption maximum in the red part of the spectrum, 660 nm LEDs offer the best technical compromise in terms of cost, efficiency and power output.

## Dehumidification

The focus thus far has been on energy for lighting but a large contributor to total energy cost which can be overlooked as it is not as visible is the dehumidification of air in VF. Water is taken up by plant roots and transpired by leaves as water vapour—an inevitable physical accompaniment to the need to take up CO_2_ from air for photosynthesis. Open stomata allow carbon dioxide into internal leaf spaces but also allow water vapour from the moist internal tissues to diffuse to the drier ambient air, causing water loss from leaves. Without the removal of transpired water vapour from the air, relative humidity would run away to near-saturation and increase the risk of fungal diseases. It would also restrict the transport within the xylem of important plant nutrients, such as calcium which is considered less mobile as it is predominantly transported through plant tissues by water flow through the plant.

Without removal of transpired water vapour from the air, relative humidity would run away to near-saturation and increase the risk of fungal diseases.

Dehumidification is achieved by condensing-coils in the air conditioning system which cool the air to below the dewpoint temperature. The condensate is then routed back to the nutrient solution which means that VF is extremely efficient in terms of water usage. However, the energy required for dehumidification is considerable though there is no general rule about how much these costs will be. The cooling needed to dehumidify the air will depend on the amount of water vapour that must be removed, and this will depend on the amount of transpiration by the plants; most of the water taken up by plants is transpired with only a small fraction being used by photosynthesis. Transpiration in turn will depend on leaf temperature, the water vapour pressure deficit between the leaf air spaces and the surrounding air (this is the driving force for water diffusion) and stomatal and boundary layer conductances to water vapour diffusion. The stomatal conductance will depend on the rate of photosynthesis and the carbon dioxide concentration in the air in the VF: the higher the latter, the lower will be stomatal conductance. Boundary layer conductance will depend on leaf and plant architecture and the speed of air movement in the vertical farm, something that depends on ventilation fans, which use electricity. Apart from evaporation from leaves, water will also evaporate from any other exposed water surface. The amount of water that needs to be removed from the air is therefore variable depending on leaf area, plant architecture, photosynthetic rate and VF environment.

The cooling system that cools and dehumidifies the air will generate a heat output but what happens to this heat will depend on the energy budget of the VF, particularly the production of heat from the lighting system and the dissipation of energy from photosynthesis. The maximum energy stored by photosynthesis is about 35% when light is supplied as red photons and assuming that photosynthesis is otherwise working perfectly with no losses—not the case in the real world. Most of the light energy that is supplied to a photosynthetic engine is therefore, even under optimal conditions, dissipated as heat, a loss that will be even greater when other limitations are present such as the loss of LUE that occurs when light intensity is higher than the strictly light-limiting range of intensities. VF systems therefore need to dissipate a lot of heat so it is not often possible to recapture and use the waste heat released by the cooling systems.

## Airflow

Airflow patterns within VFs have been the subject of numerous CFD-based studies (Sohn et al, 2023; Agati et al, 2024; Kang et al, 2024) and are surprisingly complex given the apparent geometric simplicity of a typical vertical farm. The main purpose of those studies has been to characterise the development of microclimates which would defeat the objective of having a uniform, highly controlled and accurately known set of growth conditions. Undesirable microclimates can easily go undetected depending on sensor placement, as sensors will only take a ‘point’ measurement of their immediate environment or an average measurement of different air masses which are forced to mix along return flow ducting. Plants, too, only sense their immediate environment, and themselves modify airflow greatly by producing drag. In vertical farms, airflow is usually unidirectional with generally low and constant flow rates, which differs from the varying conditions found in an open field. Low airflow can create stagnant flow patterns within the canopy, further encouraging microclimate development and even CO_2_ depletion in boundary layers around leaves. Heat produced by lighting or any other heat sources can lead to vertical thermal stratification, affecting growth rates at different levels.

It is worth noting that plants are ‘sensors’ themselves—biological ones—and a non-uniform crop could well be the result of poor climate uniformity, even when sensors may be suggesting otherwise. In the high-tech, sensor-rich environment that is the vertical farm, the reliance on sensors can itself be problematic at times. Often, a visual inspection and an eye for detail can offer more information than any sensor and is all that is needed to uncover practical anomalies such as potential climate heterogeneity.

## Start-up costs

So far, we have taken a look at the energy cost of VF but equally considerable are the start-up costs. VF has in many cases modelled itself as a high-tech, highly automated enterprise in which control and monitoring technologies are viewed as a linchpin for higher yields by providing growth conditions carefully tuned to the needs of the plant. A drawback of this approach is that it significantly increases up-front capital expenditure.

The goal of automation is to reduce labour costs, which is especially important in advanced economies. Still, there remains the need for skilled labour to maintain generally smooth operation. It could be argued that the high cost of automation, as attractive as automation itself may be, is difficult to justify in relation to generally low margins of fresh produce. Therefore, while technology can bring significant cost benefits for production, these must be carefully weighed against the product being produced and the initial investment costs. A perusal of VF operations suggests that many believe that automation gives them a competitive advantage over other forms of agriculture. Somewhat ironically, though, the result of costly automation seems to have been a high debt burden exacerbated by high energy costs, which together have, in many cases, not been offset by higher yields and lower labour costs. The level of automation and manual labour is a fine balancing act which will vary by region and crop type—some crops will lend themselves to mechanical harvesting more than others—remaining mindful that more technology does not necessarily translate into a more economically viable operation.

… while technology can bring significant cost benefits for production, these must be carefully weighed against the product being produced and the initial investment costs.

## The role of improved genetics in VF

The vertical farm is generally an alien environment for plants, given the completely artificial growth conditions. As such, VF is presented with the conundrum of having to select genotypes which excel in the alien environment but having access only to genotypes which were selected for commercial greenhouse or open-field production.

… VF is presented with the conundrum of having to select genotypes which excel in the alien environment but having access only to genotypes which were selected for commercial greenhouse or open-field production.

At best, breeding can take several years, and the market is still too small to attract dedicated breeding programs to suit the unique VF environment. In the meantime, vertical farms have leaned heavily on technological solutions to improve efficiency while using existing commercially available genotypes. There is, however, no reason to expect that genetic adaptations could not yield tremendous advances in yield as they have done in the greenhouse and open field. Ultimately, technology and genetics should complement one another in a two-pronged approach while also being mindful that some problems could better be solved genetically rather than technologically and vice versa.

The question remains as to which genetic improvements should be targeted. In a VF environment where light energy represents a substantial cost to production, the benefits of improving photosynthetic efficiency would be especially relevant and indeed this has been of much interest in crop improvement (Zhu et al, 2010; Evans, 2013; Simkin et al, 2019; Smith et al, 2023; Croce et al, 2024). However, even if photosynthetic efficiency were to be increased, the investment of biomass into harvestable plant organs—the so-called harvest index (HI)—is another crucial factor that determines what a vertical farm can grow profitably. Currently, vertical farms are largely limited to high-HI crops such as lettuce, herbs or other leafy crops. Producing staples, which typically have a considerably lower HI than leafy crops, will require substantial increases to their current HI. This means reducing allocation to non-edible plant organs or those not intended for sale; one approach could include a reduction in root biomass, since the abundance of water and nutrients in hydroponic growing systems should mean that fewer roots are needed. Root biomass could also be restricted by non-genetic, mechanical means such as air pruning. This highlights how an approach to a specific problem can be approached from a genetic or technological standpoint, or a combination of both, with the decision based on underlying economics.

The purpose of VF is typically to produce a large amount of product using a small footprint. Stacking of plants naturally allows for this but the number of layers could be increased by breeding for dwarf varieties or improving existing dwarf varieties. An example is tomato, where dwarf varieties are gaining interest for application in VF. Dwarf varieties, with their lower vertical profile, could also be mechanically handled by automation systems in the same way as leafy crops. However, self-shading in dwarf varieties can be significant given their compact growth habit, which could impact yield.

This leads to another area of potential improvement, namely improved plant architecture. An ideal architecture is one in which the ratio of projected leaf area to total leaf area is equal to unity, meaning no self-shading. Vertical farms typically tend to employ red and blue light which is very strongly absorbed by the uppermost light-exposed leaves. Most of this radiation is supplied by point-source lamps above the crop which limits lateral radiation that could penetrate more deeply within the canopy. Leaves within the canopy therefore receive very little irradiance and may be below the light compensation point, making them light sinks rather than sources. With light energy being an expensive resource, whole-plant LUE and not just leaf-level LUE is crucial to use light efficiently.

## Post-harvest considerations

The general absence of abiotic stress, pests and disease in a well-controlled vertical farm should, in practice, generate a product of excellent quality and, indeed, vertical farms often cite superior quality of fresh produce as a competitive advantage over other less-controlled modes of agriculture. Even so, high quality at harvest cannot be assumed to translate to high post-harvest quality. In fact, circumstances can be envisaged under which these two parameters could diverge. For example, a high-growth humidity—which would be desirable since dehumidification carries significant energy costs—has been shown to create non-responsive stomata which do not close upon interaction with drier environments (Fanourakis et al, 2020). The resulting water loss causes the product to quickly lose its firmness and crunch, both of which are valued by consumers as hallmarks of freshness.

… high quality at harvest cannot be assumed to translate to high post-harvest quality.

Often, if not always, absent in VF light spectra is UV light. It may seem counterintuitive to add this abiotic stress to an artificial environment. However, a multitude of benefits have been associated with UV light. It stimulates thicker cuticles in leaves which, while not especially relevant during growth, could make harvested product more resistant to blemishing from handling or reduce water loss (Lara et al, 2019). Somewhat ironically, therefore, some stressors may be beneficial when considering post-harvest characteristics rather than simply aiming to maximise harvestable fresh weight. Other benefits of UV light are that it stimulates the synthesis of secondary metabolites which can impart desirable attributes such as colouration—for example, the purple colouration of red lettuce varieties caused by anthocyanins—or unique flavour profiles. Other parts of the spectrum have also been shown to impact post-harvest quality, nutrient profile, flavour and/or colouration in lettuce, basil and arugula (Nicole et al, 2019).

## Looking to the future

The advantages associated with VF are lower water, fertiliser and pesticide usage as well as shorter transport distances of the product. Often, these factors are put forward as reasons why vertical farming *ought* to exist but none of these purported environmental benefits on their own, as attractive as they may be, lead to VF becoming more than a fringe agricultural production system. This is because the environmental benefits of vertical farming do not automatically translate into profits. While the consumer may appreciate the environmental friendliness of VF, they may not be prepared to pay higher costs for fresh produce, especially at a time when inflation puts stress on consumers to save money.

Can VF become truly global and is it the panacea to the challenge of producing sufficient food in a sustainable manner? Some qualification is needed here: while lettuce or herbs are regarded as food, they do not meaningfully contribute to daily total caloric intake though they can form part of a healthy diet as a source of vitamins and minerals. Truly tackling growing food demand would require that VF produce protein- or carbohydrate-rich staples like grains, legumes or potatoes. To gain mainstream adoption, VF will need to compete with conventional agriculture on quality *and* price. Competing on price will depend largely on labour and energy prices which are typically high in the developed world where, currently, most vertical farms are located. Beneficially, however, selling prices are typically also higher in such regions.

Truly tackling growing food demand would require that VF produce protein- or carbohydrate-rich staples like grains, legumes or potato.

At the same time, vertical farms will also need to confront and address their copious use of energy when nuclear and fossil fuels remain the predominant energy sources. Even if—hypothetically at this stage—VF were to be powered entirely by renewable sources, the question arises as to whether that energy should be used for VF or elsewhere. As with all attempts to solve environmental challenges, great care should be taken to not shift a problem elsewhere. From a business sustainability perspective, the sensitivity of the cost of production to energy price fluctuations represents a serious risk for long-term financial sustainability. An example was the severe negative impact of the Ukraine war on European energy prices which put significant pressure on vertical farms across Europe.

… vertical farms will also need to confront and address their copious use of energy when nuclear and fossil fuels remain the predominant energy sources.

A moral dilemma also emerges from where VF can realistically be established in the first place. Shipping VF-grown produce globally directly contradicts its *raison d*’*etre* so produce must be produced locally. However, the extensive infrastructure and skills needed to establish and operate a vertical farm are absent or inadequate in much of the developing world, precisely where population malnutrition is prevalent. In many developing countries, electricity supply is often unreliable, and in any case the market for premium VF-grown fresh produce is likely diminishingly small amongst a generally low-income consumer base.

Despite the many multidisciplinary challenges facing VF, it is important to explore how it could contribute to shortcomings in global food production. Adequate food production in a sustainable manner, together with food security, are some of the most pervasive challenges of our time, which will demand innovative and tangible shifts to old paradigms. Vertical farms are an attempt to achieve this, emerging at a time when threats to, and pressure on arable land by climate change, urbanisation, land degradation and population growth are unprecedented. It is a matter of when—rather than if—food production will become an even more pressing problem than it presently is. In some ways, VF may be ahead of its time, though it is foreseeable that a tipping point is looming at which, either through continued land degradation and/or government policy, it may become a far more viable means of food production. At that point, the economies of scale of mass-produced, more standardised, modular vertical farms would significantly reduce start-up costs, helping to pave the way for widespread implementation. If, for whatever reason, VF fails to find mainstream adoption on our own planet, the technology will undoubtedly be crucial for the increasingly likely prospect of extra-planetary human settlement.

In some ways, VF may be ahead of its time, though it is foreseeable that a tipping point is looming at which […] it may become a far more viable means of food production.

## Supplementary information

Peer Review File
