# Supplementary material for: Food for thought: Perspectives on the current state of Vertical Farming
Source: EMBO Rep. 2025 Jul 21;26(16):3982–90. doi: 10.1038/s44319-025-00518-1 (PMC12373847; doi:10.1038/s44319-025-00518-1)
Supplement: Supplementary file 1 — Peer Review File [file 44319_2025_518_MOESM1_ESM.pdf]

Food for thought; Perspectives on the current state of Vertical Farming

Jeremy Harbinson and Craig Taylor

Corresponding author: Jeremy Harbinson (Jeremy.Harbinson@wur.nl)

Review Timeline:

Submission Date:	23rd Jun 25
Accepted:	25th Jun 25

Editor: Holger Breithaupt

Transaction Report:

This article was editorially reviewed.
